# Implicit expression of uncertainty in medical students during different sequences of clinical reasoning in simulated patient handovers

**DOI:** 10.3205/zma001589

**Published:** 2023-02-15

**Authors:** Sigrid Harendza, Hans Jakob Bacher, Pascal O. Berberat, Martina Kadmon, Julia Gärtner

**Affiliations:** 1 Universitätsklinikum Hamburg-Eppendorf, III. Medizinische Klinik, Hamburg, Germany; 2 Technische Universität München, Fakultät für Medizin, TUM Medical Education Center, München, Germany; 3 Universität Augsburg, Medizinische Fakultät, Dekanat, Augsburg, Germany

**Keywords:** assessment, communication, competence, simulation, handover, uncertainty

## Abstract

**Background:**

Dealing with medical uncertainty is an essential competence of physicians. During handovers, communication of uncertainty is important for patient safety, but is often not explicitly expressed and can hamper medical decisions. This study examines medical students’ implicit expression of uncertainty in different sequences of clinical reasoning during simulated patient handovers.

**Methods:**

In 2018, eighty-seven final-year medical students participated in handovers of three simulated patient cases, which were videotaped and transcribed verbatim. Sequences of clinical reasoning and language references to implicit uncertainty that attenuate and strengthen information based on a framework were identified, categorized, and analyzed with chi-square goodness-of-fit tests.

**Results:**

A total of 6358 sequences of clinical reasoning were associated with the four main categories *“statement”, “assessment”, “consideration”*, and *“implication”*, with statements occurring significantly (p<0.001) most frequently. Attenuated sequences of clinical reasoning occurred significantly (p<0.003) more frequently than strengthened sequences. Implications were significantly more often attenuated than strengthened (p<0.003). Statements regarding results occurred significantly more often plain or strengthened than statements regarding actions (p<0.0025).

**Conclusion:**

Implicit expressions of uncertainty in simulated medical students’ handovers occur in different degrees during clinical reasoning. These findings could contribute to courses on clinical case presentations by including linguistic terms and implicit expressions of uncertainty and making them explicit.

## Zusammenfassung

**Zielsetzung:**

Der Umgang mit medizinischer Unsicherheit ist eine wesentliche Kompetenz von Ärzt*innen. Bei Übergaben ist die Kommunikation von Unsicherheit wichtig für die Patient*innensicherheit, wird jedoch oft nicht explizit ausgedrückt und kann medizinische Entscheidungen behindern. Diese Studie untersucht den impliziten Ausdruck von Unsicherheit bei Medizinstudierenden in verschiedenen Sequenzen des klinischen Argumentierens während simulierter Patient*innenübergaben.

**Methodik:**

Siebenundachtzig Medizinstudierende im letzten Studienjahr nahmen im Jahr 2018 an Übergaben von drei simulierten Patient*innenfällen teil, die videografiert und wörtlich transkribiert wurden. Sequenzen des klinischen Argumentierens sowie Sprachverweise auf implizite Unsicherheit in Form informationsabschwächender und -verstärkender Ausdrücke wurden basierend auf einem Rahmenwerk identifiziert, kategorisiert und mit Chi-Quadrat-Anpassungstests ausgewertet.

**Ergebnisse:**

Insgesamt waren 6358 Sequenzen des klinischen Argumentierens den vier Hauptkategorien „Bericht“, „Bewertung“, „Erwägung“ und „Schlussfolgerung“ zuzuordnen, wobei Berichte signifikant (*p*<0,001) am häufigsten vorkamen. Abgeschwächte Sequenzen klinischen Argumentierens kamen signifikant (*p*<0,003) häufiger vor als verstärkte Sequenzen. Schlussfolgerungen waren dabei signifikant häufiger abgeschwächt als verstärkt (*p*<0,003). Bei Berichten waren Ergebnisse signifikant häufiger neutral oder verstärkt als Maßnahmen (*p*<0,0025).

**Schlussfolgerung:**

Impliziter Ausdruck von Unsicherheit kommt in simulierten Übergabegesprächen von Medizinstudierenden in unterschiedlicher Ausprägung beim klinischen Argumentieren vor. Diese Befunde könnten dazu beitragen, in Kursen zu klinischen Fallvorstellungen sprachliche Äußerungen und implizite Ausdrücke von Unsicherheit einzubeziehen und explizit zu machen.

## 1. Introduction

In their undergraduate and postgraduate training, physicians acquire the ability to make judgments that they use in their everyday work, which is characterized by medical uncertainty, in order to reason clinically [1]. Medical uncertainty includes both, patient-related factors such as medical history, test variability or diversity of information sources as well as physician-related factors such as communication quality, test interpretation and uncertainty tolerance [2]. Each patient contact requires a cognitive decision-making process consisting of information acquisition, hypothesis generation, and derivation of diagnostic and therapeutic steps [3]. This process is the basis of clinical reasoning [3]. These aspects mentioned above are summarized in focused case presentations during patient handovers to ensure correct medical patient care [4]. In many cases, medical uncertainty is not openly communicated [5]. The reasons for this behavior can be found in medical socialization, where correct knowledge and good grades are held in high esteem above all other aspects [6], [7], [8]. The expression of medical uncertainty tends to be associated with shame, as the disclosure of medical uncertainty is often interpreted as a lack of competence [9], [10] and is thus also associated with one's own vulnerability in professional action [8]. Medical students even tend to mask uncertainty [9].

Low tolerance of uncertainty can impede medical decision-making [11]. The accompanying communication errors can lead to unnecessary hospital admissions, can trigger unnecessary diagnostics requests, and can compromise patient safety [12], [13], [14]. In a previous study [15], we elaborated the identification and distribution of implicit linguistic expressions of medical uncertainty related to patient cases (i.e., the patient-related factor of medical uncertainty [2]). Based on the same data, the aim of the current study was the communication of implicit uncertainty in the context of clinical reasoning (i.e., the physician-related factor of medical uncertainty [2]).

## 2. Methods

### 2.1. Study design and participants

In 2018, 87 final-year medical students (67.4% female, 32.6% male) from three medical faculties (Hamburg, Oldenburg, TU Munich) voluntarily participated in a competence-based assessment simulating the first day of residency [16]. The assessment included a consultation hour with three simulated patients per participant, who were handed over to other participants after collection of additional information. The patients, based on real cases from the emergency department, were portrayed by professional, trained actors and actresses. The cases were based on either a chief complaint or a chief finding (e.g., man with very severe abdominal pain: abdominal migraine; woman with elevated creatinine level: acute renal failure due to hantavirus). A detailed description of the patient cases can be found in Gärtner et al. [15]. The cases were chosen in a way that they could only be solved by analytical thinking and not by pattern recognition alone [17]. At the end of their shift, the medical students handed three cases over to a peer who did not know these cases and discussed further diagnostics and therapy. The handovers were videotaped.

#### 2.2. Instruments

To analyze implicit uncertainty in students during the patient handovers, we used an empirically derived framework [18]. It includes four main categories, which are each represented by a textual sample: *“statement”* (“I have done a urine dipstick.”), *“assessment”* (“Stool and urine were unremarkable.”) *“consideration”* (“[A] kidney biopsy can also be considered.”), and *“implication”* (“At some point later [...] we should do a TTE.”). In each of these main categories, the subcategories* “action”* (“[...] we will ask for a small blood count and an ECG.”) and *“result”* (“[...] no known pre-existing conditions.”) occur. In addition, the framework includes four types of linguistically modifying expressions, each of which either attenuates information (e.g., “maybe”, “doubtful”, “probably”) and thus implicitly refers to increased uncertainty (*“attenuated”*), or strengthens information (e.g., “definitely”, “of course”, “absolutely”) and thus implicitly refers to decreased uncertainty (*“strengthened”*). Furthermore, statements without these strengthening or attenuating modifiers (*“plain”*) were found as well as statements accompanied by both, attenuating and strengthening modifiers (*“mixed*”). A detailed description of the linguistic expressions used and their subcategories can be found in Gärtner et al. [18].

#### 2.3. Data analysis

The videotaped patient handovers were transcribed verbatim. Using MAXQDA Analytics Pro 2020 (Release 20.0.8, VERBI GmbH), we assigned the sequences to the four main categories and the respective subcategory. We then searched for the framework’s linguistic expressions that implicitly attenuated or strengthened information [18]. All expressions that did not literally address uncertainty were understood as implicit in this study (e.g., “I am uncertain”=explicit; “I don't know”=implicit). Possible differences in the distribution of statements across the four main categories and the three modifiers “attenuated”, “plain”, and “strengthened” were calculated using chi-square goodness-of-fit tests. The significance level was set at p<0.05 and set at 0.003 using the Bonferroni correction for multiple testing of 15 comparisons. The distribution of modifiers across the subcategories “action” and “result” was also analyzed using chi-square adjustment tests, and the Bonferroni-corrected significance level was set at p<0.0025 based on 20 comparisons.

## 3. Results

In total, 6358 statements could be found and assigned to one of the four main categories that resemble sequences of clinical reasoning (*“statement”, “assessment”, “consideration”, “implication”*), with “statement” being significantly (p<0.001) the most frequent (see table 1 (Tab. 1)). Overall, attenuated sequences of clinical reasoning were significantly (p<0.003) more frequent than strengthened sequences. For *“statement”, “implication”*, and *“assessment”*, plain information occurred significantly most frequently (p<0.003). Implications were significantly more frequently attenuated than strengthened (p<0.003), while statements were significantly more frequently strengthened than attenuated (p<0.003).

Table 2 (Tab. 2) shows the ratios of actions and results within the main categories of the clinical reasoning sequences and modifiers. While actions occurred significantly more frequent for *“implication”* than results, this was reversed for* “statement”* and *“assessment”* (p<0.0025). For *“statement”*, results were significantly more likely to be plain or strengthened than actions (p<0.0025); for attenuated statements, there was no significant difference between results and actions. For *“assessment”*, results were significantly (p<0.0025) more likely to be attenuated, plain, or strengthened than actions, while for *“implication”*, actions were significantly (p<0.0025) more likely to be attenuated, plain, or strengthened than results.

## 4. Discussion and conclusion

Plain statements occurred most frequently in the handovers, whereby stated results included significantly more implicit references to decreased uncertainty and stated actions included more implicit references to increased uncertainty. Implications, which are of particular importance in clinical reasoning, showed the highest amount of implicit references to increased uncertainty. If this uncertainty is not noticed by the persons receiving a handover, it may contribute to treatment errors in some instances. Stated actions as well as considerations and implications play a crucial role in weighting diverging hypotheses for further patient treatment. Therefore, uncertainty should be recognized and explicitly expressed in undergraduate and postgraduate training, especially in the context of patient presentations during handovers [19]. The SNAPPS technique (S: summarize history and findings, N: narrow the differential, A: analyze the differential comparing and contrasting the possibilities, P: probe preceptors about uncertainties, P: plan management, S: select case-related issues for self-study) could be shown to support students in perceiving their uncertainty [20]. Moreover, medical students who had attended a handover course or a clinical reasoning course were able to hand over patients in a more focused manner [21], [22]. Our findings on sequences of clinical reasoning and linguistic expressions that implicitly express uncertainty could add to focusing on these linguistic expressions in such courses. This could be used to design exercises on how to make perceived implicit uncertainty explicit and discussable to improve patient safety. Recorded handovers, for example, could be watched in communication courses and, using the framework, implicit expressions of uncertainty could be explored [18]. In a next step, medical students could consider how to express uncertainty explicitly. The effect of such reflections could be explored at the end of such a course by conducting handovers again with a focus on changes in the linguistic characteristics.

## Funding

This study is part of the ÄKHOM project, funded by the German Federal Ministry of Education and Research (BMBF), grant number: 01PK1501A/B/C. Recipients of this funding were SH, POB and MK. The funders had no influence on the study design, data collection and analysis, decision to publish, or preparation of the manuscript.

## Ethics

The Ethics Committee of the Hamburg Medical Association approved the study including written informed consent of participants and anonymized and voluntary participation (reference number: PV3649).

## Acknowledgement

We would like to thank all the medical students who participated in this study. We thank Dr. T. Urbanowicz for her assistance in preparing the transcripts.

## Competing interests

The authors declare that they have no competing interests. 

## Figures and Tables

**Table 1:**
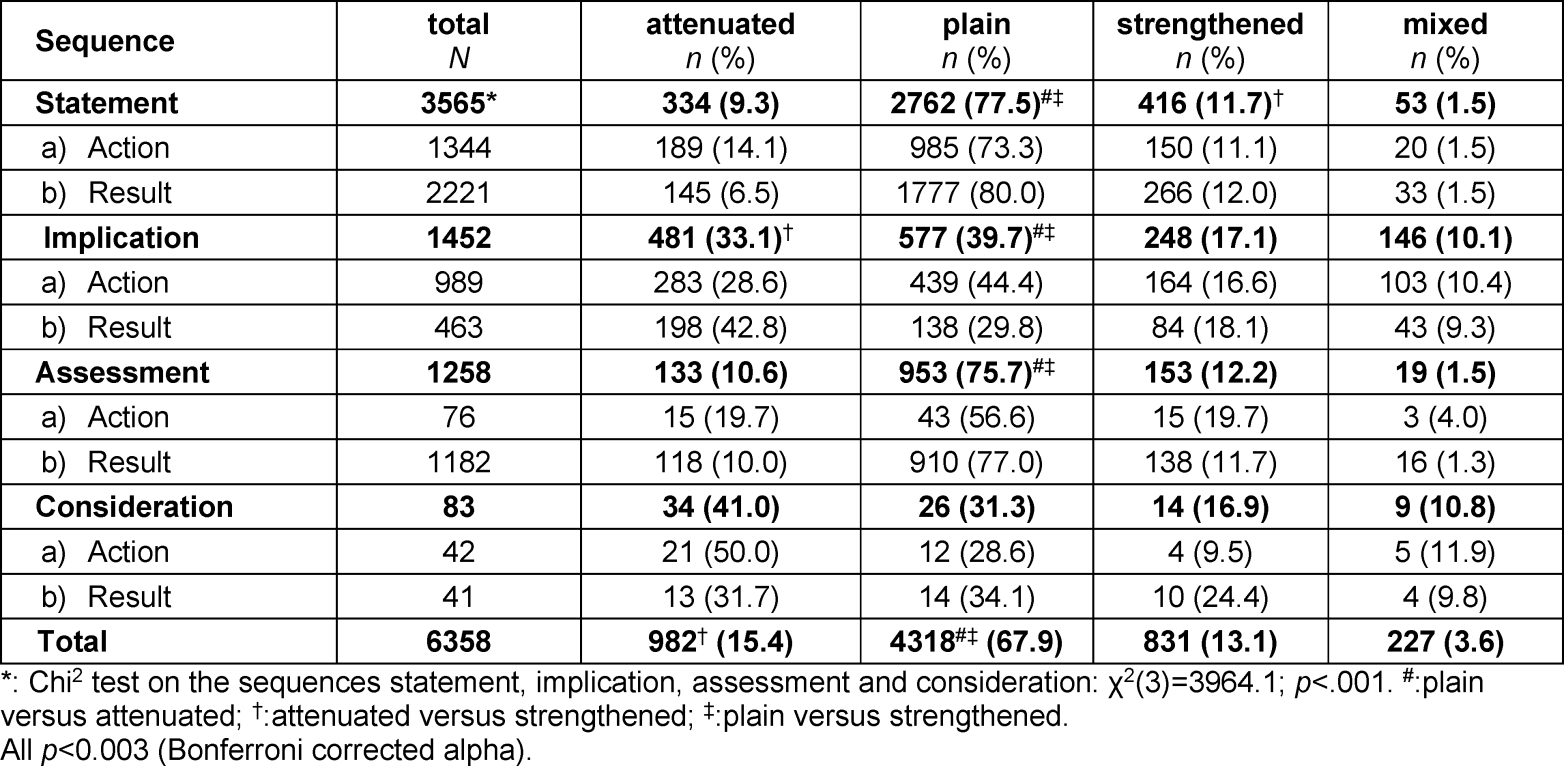
Frequencies of modified sequences of clinical reasoning

**Table 2:**
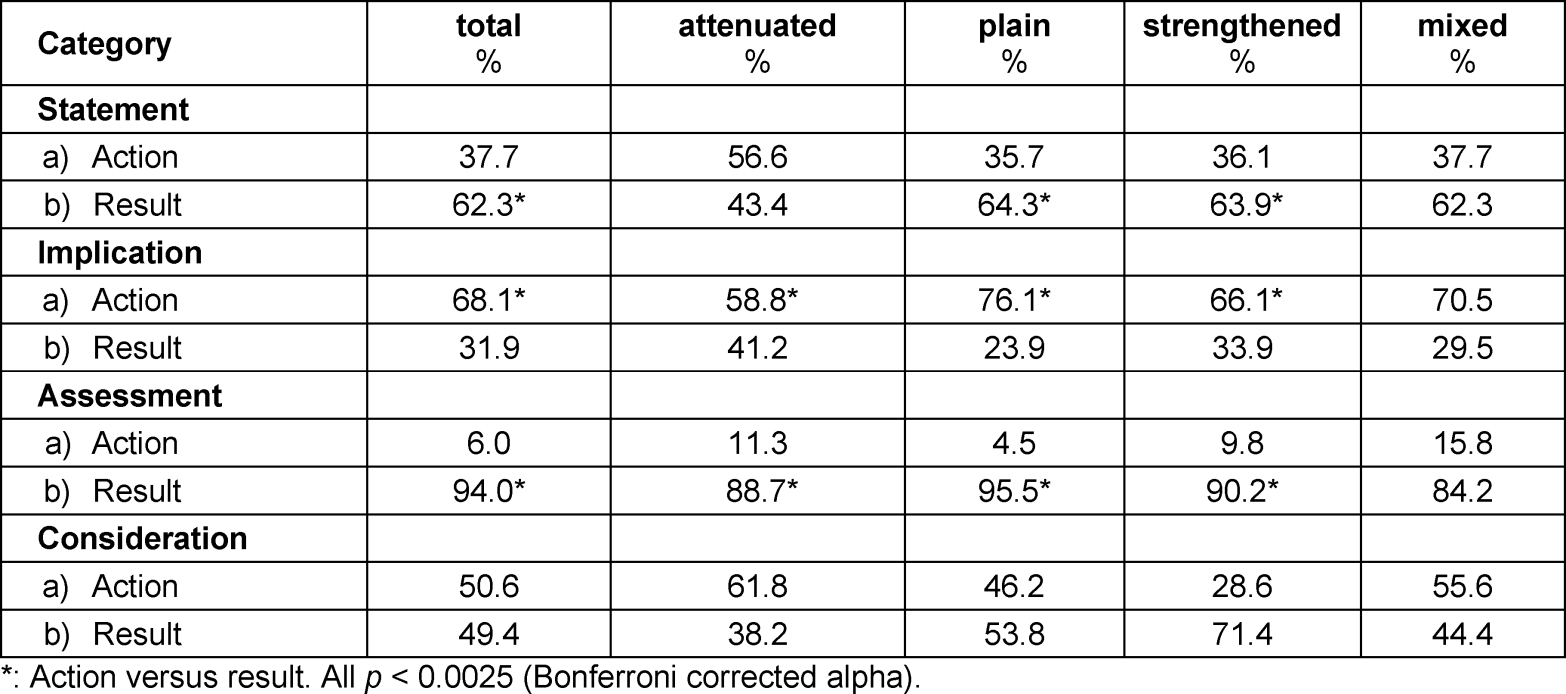
Percentage of action und result in the categories of the modifiers
